# Atherogenic, fibrotic and glucose utilising actions of glucokinase activators on vascular endothelium and smooth muscle

**DOI:** 10.1186/1475-2840-13-80

**Published:** 2014-04-15

**Authors:** Sefaa Al-aryahi, Danielle Kamato, Robel Getachew, Wenhua Zheng, Simon J Potocnik, Neale Cohen, Daniel Guidone, Narin Osman, Peter J Little

**Affiliations:** 1 Discipline of Pharmacy and Diabetes Complications Group, Health Innovations Research Institute, School of Medical Sciences, RMIT University, Bundoora, VIC 3083, Australia; 2 State Key Laboratory of Ophthalmology, Zhongshan Ophthalmic Center, Sun Yat-sen University, Guangzhou, China; 3 Discipline of Cell Biology and Anatomy, School of Medical Sciences, RMIT University, Bundoora, VIC 3083, Australia; 4 Diabetes Clinical Services, BakerIDI Heart and Diabetes Institute, Melbourne, VIC 3004, Australia; 5 Departments of Medicine, Nursing and Health Sciences and Immunology, Monash University School of Medicine (Central and Eastern Clinical School, Alfred Health), Prahran, VIC 3004, Australia

**Keywords:** Transforming growth factor beta, Smads, Diabetes, Pleiotropic actions

## Abstract

**Background:**

Pharmaceutical interventions for diabetes aim to control glycaemia and to prevent the development of complications, such as cardiovascular diseases. Some anti-hyperglycaemic drugs have been found to have adverse cardiovascular effects in their own right, limiting their therapeutic role. Glucokinase activity in the pancreas is critical in enhancing insulin release in response to hyperglycaemia. Glucokinase activators (GKAs) are novel agents for diabetes which act by enhancing the formation of glucose-6-phosphate leading to increased insulin production and subsequent suppression of blood glucose. Little, however, is known about the direct effects of GKAs on cardiovascular cells.

**Methods:**

The effect of the GKAs RO28-1675 and Compound A on glucose utilisation in bovine aortic endothelial cells (BAEC) and rat MIN6 was observed by culturing the cells at high and low glucose concentration in the presence and absence of the GKAs and measuring glucose consumption. The effect of RO28-1675 at various concentrations on glucose-dependent signalling in BAEC was observed by measuring Smad2 phosphorylation by Western blotting. The effect of RO28-1675 on TGF-β stimulated proteoglycan synthesis was measured by ^35^S-SO_4_ incorporation and assessment of proteoglycan size by SDS-PAGE. The effects of RO28-1675 on TGF-β mediated Smad2C phosphorylation in BAEC was observed by measurement of pSmad2C levels. The direct actions of RO28-1675 on vascular reactivity were observed by measuring arteriole tone and lumen diameter.

**Results:**

GKAs were demonstrated to increase glucose utilisation in pancreatic but not endothelial cells. Glucose-activated Smad2 phosphorylation was decreased in a dose-dependent fashion in the presence of RO28-1675. No effect of RO28-1675 was observed on TGF-β stimulated proteoglycan production. RO28-1675 caused a modest dilation in arteriole but not contractile sensitivity.

**Conclusions:**

GKA RO28-1675 did not increase glucose consumption in endothelial cells indicating the absence of glucokinase in those cells. No direct deleterious actions, in terms of atherogenic changes or excessive vasoactive effects were seen on cells or vessels of the cardiovascular system in response to GKAs. If reflected *in vivo*, these drugs are unlikely to have their use compromised by direct cardiovascular toxicity.

## Background

There is an epidemic of Type 2 diabetes mostly driven by increasing worldwide obesity and sedentary life style factors [1,2]. The defining metabolic parameters of Type 2 diabetes are insulin resistance and pancreatic beta cell failure and they are associated with increased rates of cardiovascular disease [3-5]. Existing drugs for the treatment of these factors have thus far been overwhelmed by the pervasive natural history of the disease and no treatment regimens are available which can maintain long term normoglycemia and prevent cardiovascular disease.

The rapid increase in the occurrence of obesity-related Type 2 diabetes commenced in the early 1980s [6]. This event was not fully appreciated by the pharmaceutical industry which in the relevant period and area was concentrating on the development of anti-hypertensive agents; accordingly, there was a large gap in the discovery and introduction of new classes of drugs for the treatment of hyperglycemia. Intense research over the last several decades has partially redressed this situation and there are now many new and emerging classes of anti-hyperglycemia agents including, incretin mimetics, dipeptidyl peptidase (DDP)-4 inhibitors, sodium glucose co-transport 2 inhibitors and in the current context, glucokinase activators (GKAs) [7-9]. Glucokinase activators (GKAs) are at different stages of clinical development [10]. GKAs have been under clinical development since 1990 and in this time over 100 patent applications for this class of compound have been filed, with several agents proceeding to short-term clinical trials in humans [11]. The large number of GKAs available and chemical diversity amongst these molecules makes generalisation and intra-class comparison difficult [12]. Broadly, the class shows high effectiveness at reducing fasting and basal glucose levels and improves glucose tolerance [11]. GKAs have effects at the liver which may be beneficial in reducing hepatic glucose output, but may have the unwanted effect of inducing hypertriglyceridemia and hepatic steatosis [12,13]. Recent studies with a GKA have shown that in rats one analogue enhances beta cell function and suppresses hepatic glucose production, both essential elements of an anti-hyperglycaemic action [14]. The cardiovascular risk associated with these effects warrants investigation notwithstanding that it must be appreciated that there is currently no GKA in current clinical use.

Hexokinases (HK) are enzymes that utilise phosphate from ATP to phosphorylate hexoses yielding phosphohexose and thus, catalysing the conversion of glucose to glucose-6-phosphate (G-6-P). There are four mammalian HK isozymes - Type I, II, III and IV that are located in different tissues of the body and they differ in their physiological function [15]. HKI-III are 100 kDa proteins with relatively high affinity for glucose and are inhibited by physiological concentrations of glucose-6-phosphate (G-6-P) [15,16]. HKI and III have a catalytic active site at the carboxyl terminal region whereas HKII has a catalytic active site in both the amino (N) and the carboxyl (C) terminal [15]. HKI is found in high levels in the brain and skeletal muscle, HKII is found in insulin-sensitive tissues including skeletal muscle, adipose tissue and vascular smooth muscle cells.

HKIII is found in the kidney, lung, liver, spleen and brain [15]. In the family of hexokinases, glucokinase (GK), also known as hexokinase IV or D, has a lower affinity for glucose than other members of the hexokinase family; GK responds to and regulates glucose concentrations at higher glucose levels than the other isoforms. GK is less inhibited by its catalytic product, glucose-6-phosphate (G-6-P) than are the other members of the HK family. GK senses (blood) glucose in the physiological level and higher, hence pathophysiological range, and its action leads to the enhanced secretion of insulin from pancreatic beta cells. The expression of GK has not been thoroughly investigated and it is presently considered to have a narrow spectrum of tissue expression mostly in glucose sensing and sensitive tissues (pancreas and liver).

Physiologically, increasing blood glucose levels, for example, post-prandially, leads to activation of GK, increased insulin secretion and a blood glucose lowering response [17]. With the knowledge that genetic mutations of GK had effects on glucose metabolism, the potential arose that GKs could be targeted therapeutically for the regulation of glucose metabolism and potentially for the treatment of the hyperglycaemia of diabetes [18]. Drug development in this area has yielded several structurally distinct classes of agents with differing effects and potencies [19,20]. Of the family of HKs I – IV, GK is the only isoform with an “activator site” and thus the only HK which is theoretically mechanistically responsive to GKAs. GKAs target an allosteric site that is only exposed when glucose is bound to GK and GKAs interact with this site in a thermodynamically reversible manner. The binding of a GKA to the allosteric site of a glucose bound GK enzyme stabilizes the GK in an active conformation which activates the enzyme and also prevents its interaction with glucokinase regulatory protein (GKRP) and prevents nuclear sequestration. There is no published data showing GKAs effects on other HKs except for two references to unpublished data in papers from pharmaceutical company laboratories which state GKAs were specific for GK and do not activate HKI-III [21,22]. The *in vivo* actions (hypoglycaemia) of most GKAs correlate closely with their *in vitro* efficacies which indicates that the proposed biochemical action, being activation of GK, is the actual mechanism of action mediating the hypoglycaemic effects *in vivo*[23]. There are also other drugs that may work at least partially via an action involving GKs – mitiglinide induces translocation of GK from the nucleus to the cytoplasm where it can mediate a hypoglycaemic effect [24,25].

As the history of drug development in the area of diabetes and cardiovascular disease well reflects [26-28], efficacy is assessed as the effect on a risk factor but the ultimate target of a particular therapy, the effect on CVD, is often not considered. It is possible to hypothesize that if glucose causes cardiovascular disease and GKAs stimulate the entry and utilization of glucose by cells of the vascular system, then GKAs might indeed have direct deleterious effects on the cardiovascular system. These effects might arise from either the presence of GKAs in cardiovascular cells or nominally off target effects of GKAs to activate GKs in vascular smooth muscle and endothelial cells.

We have previously examined the effects of anti-diabetic agents on atherogenic and fibrotic responses in vascular cells and multiple agents have diverse actions which contribute to our understanding of their actions [29]. We proposed a series of studies to investigate the mechanism of glucose utilisation in endothelial cells (ECs), the impact of GKAs on glucose metabolism and the vascular response to the anti-hyperglycaemic action of GKAs, and studies examining the effect of GKAs on the development of atherosclerosis in our well established *in vitro* model. The overall aim was to develop a comprehensive knowledge of factors affecting glucose effects in vascular cells and to determine if GKAs have direct or indirect hypoglycaemic actions to modify the progression of atherosclerosis. We explored the physiological connection between the effects of glucose on cell metabolism, TGF-β signalling and several other vascular properties which would reflect upon the positive or negative impact of GKAs on elements of cardiovascular disease. We observed that GKAs did not increase glucose uptake in endothelial cells and did not have pleiotropic actions to enhance glucose mediated toxicity and we identified several favourable actions most likely unrelated to actions on GK.

## Materials and methods

### Materials

Dulbecco’s Modified Eagle medium (DMEM) (0 mM and 25 mM glucose) was from GIBCO BRL, Grand Island, USA. SB431542 and DEAE-sephacel were purchased from Sigma-Aldrich, MO, USA. GKA (RO28-1675) was from Axon Medchem, The Netherlands. Anti-rabbit IgG HRP, GAPDH, anti-phospho-Smad2 (Ser465/467) rabbit monoclonal antibody and human transforming growth factor beta-1 (TGF-β) was from Cell Signalling Technology, Danvers, USA. Glucokinase activator Compound A was from Merck, Darmstadt, Germany. [^35^S]-sulfate was from MP Biomedicals, Irvine, CA. Cetylpyidinium chloride (CPC) was from Uni-Lab Chemicals and Pharmaceuticals, India. 3MM Whatman chromatography paper was from Whatman International, Ltd., Maldstone, UK. YSI 2300 Stat plus glucose and lactate analyser kindly provided by Professor Stephen Bird, Exercise and Metabolism Research Laboratory, RMIT University. YSI buffer concentrated kit, YSI glucose standard were from YSI Inc, Yellow Spring, USA.

### Culture of bovine aortic endothelial cells and rat MIN6 pancreatic beta cells

Primary cultured bovine aortic endothelial cells (BAEC) were prepared by collagenase treatments of the aortas acquired aseptically from the abattoirs [30]. The cells were passaged to provide sufficient cells for frozen stocks and experimentation. Stock cultures were thawed from the frozen stocks in liquid nitrogen and were maintained in Dulbecco’s Modified Eagle Medium (DMEM) 5 mM glucose, 10% foetal bovine serum (FBS) and 1% antibiotics (streptomycin and penicillin) and incubated in 5% CO_2_ at 37°C.

MIN6 pancreatic beta cells were provided by Professor Jun-ichi Miyazaki, Osaka University Medical School Japan. The cells were maintained in high glucose 25 mM DMEM containing 10% FBS (FBS heat inactivated for 30 min at 60°C) and 2.5 μl of 2-mercaptoethanol.

### Cell culture protocol for experimentation

For experimentation, BAEC between passages 20–50 were subcultured in 60 mm diameter dishes and 24 well plates at a density of 200,000 and 50,000 cells/well until they were confluent cultures. Cells were serum deprived in DMEM, 5 mM glucose, 0% FBS for 24 h. For glucose utilisation experiments quiescent BAEC were treated with different glucose medium concentration 2.5, 5, 15, 25 mM containing 0% FBS in the presence and absence of glucokinase activator (GKA) RO28-1675 (10 μM) and with TGF-β (2 ng/mL) as positive control and medium was collect at different time points 0, 4, 8, 24 h. For phosphorylated Smad2C protein detection experiments, BAEC were treated with RO28-1675 (0.1, 0.3, 1, 3, 10 μM) in the presence of high glucose (15 mM) for 1 h. For a time course BAEC were treated with 15 mM glucose medium at different time points 0, 15, 30, 60, 120, 240 min. For GKA dose response on phosphorylated Smad, BAEC were treated with different glucose medium concentrations 5, 10, 15, 20, 25 mM containing 0% FBS. For proteoglycan experiments cells were treated with GKA RO28-1675 (1, 3, 10 μM), SB431542 (3 μM) and GKA, Compound A (1, 3, 10 μM) in presence of TGF-β (2 ng/ml) and then radiolabeled with [^35^S]-sulfate (50 μCi/ml) for 24 hours.

MIN6 pancreatic beta cells were used as positive controls for glucose and GKA responses. These cells were subcultured between passages 30–45 in 24 well plates until they reached 80% confluence. MIN6 cells were then washed twice with DMEM containing no glucose and glucose deprived for 24 hours in DMEM containing no glucose, 10% FBS and 2.5 μl of 2-mercaptoethanol. Cells were then treated with either 5 or 25 mM glucose in the presence and the absence of two GKA compounds, RO28-1675 (10 μM) and Compound A (10 μM).

### Glucose utilisation studies

To determine cellular glucose consumption, glucose concentrations in the medium were measured pre- and post-treatment using YSI Stat plus 2300 glucose and lactate analyser. Confluent cultures were treated as described, incubated at 37°C then the media (500 μl) from each well of each treatment was collected in 1 ml eppendorf tubes and centrifuged for 10 min at 5000 rpm. Glucose concentrations were measured by placing the eppendorf tube in the sipper tube of the YSI analyser; the sipper automatically aspirates 25 μl of the sample. Glucose concentration measurements were displayed on screen. The YSI system buffer in the instrument contained sodium dihydrogen phosphate (25 mM), sodium hydrogen phosphate (45 mM), and sodium chloride (31 mM) used to flush the sample chamber through the system.

### Western blotting

Total cells lysates were resolved on 10% acrylamide gels by sodium dodecyl sulfate polyacrylamide gel electrophoresis (SDS-PAGE). Protein was then transferred to a PVDF membrane in transfer buffer (3.7% SDS, 20% methanol, 48 mM Tris base, 39 mM Glycine) at 4°C. Membranes were blocked in 5% skim milk powder and then incubated with primary rabbit monoclonal antibodies (anti-phospho-Smad2C (Ser465/467) (1:1000), anti-Smad2 (1:1000) and anti-GAPDH (1:4000)) followed by a secondary horseradish peroxidase-anti-rabbit IgG (1:1000) and ECL detection. Blots were imaged using the Bio-Rad gel documentation system and densitometry analysis was performed on blots from at least three experiments with Quantity One imaging software.

### Quantitation of proteoglycans synthesis

After cells were treated as mentioned in tissue culture for experimentation section, media from each of 24 wells containing secreted proteoglycans was collected and protease inhibitors (100 mM 6-amino caproic acid, 5 mM benzamidine hydrochloride) added. Radiolabel incorporation into proteoglycans were measured by cetylpyridium chloride (CPC) precipitation assay as previously described in detail [31].

### Assessing proteoglycan size by SDS-PAGE

Proteoglycans labelled with [^35^S]-sulfate were prepared for SDS-PAGE by isolation through the DEAE-sephacel anionic exchange mini columns. Samples were added to pre-equilibrated columns and then washed extensively with low salt buffer (8 M urea, 0.25 M NaCl, 2 mM disodium EDTA, 0.5% Triton X-100). Proteoglycans were eluted using high salt buffer (8 M urea, 3 M NaCl, 0.02 M EDTA, Triton-X).

Equal counts of proteoglycans (20,000 - 50,000 cpm) were precipitated by ethanol solution (1.3% potassium acetate in 95% ethanol) chondroitin sulfate was added as a cold carrier. Samples were suspended in 20 μl of buffer (8 M urea, 2 mM disodium EDTA, at pH 7.5) and 20 μl sample buffer (0.5 M Tris–HCl pH 6.8, 10% SDS, 50% glycerol, 2-mercaptoethanol, and 0.1% bromophenol blue). Proteoglycans were separated on gradient separating gel [32] with 4-13% acrylamide separating gels and 3% acrylamide stacking gels and run overnight at 60 V. Methylated protein molecular weight marker (Rainbow™ [^14^C]) was used. Processed and dried gels were exposed to an imaging plate (Fujifilm BAS-MS 2040 imaging plate) for approximately 4 days. Images were developed on a phosphoimager (Fujix BAS 1000 image plate scanner) and viewed using imaging software (Fujifilm Multi-Gauge).

### Cannulated arteriole assessment of vascular reactivity

All animal procedures were approved by the Animal Experimentation Ethics Committee of RMIT University. Adult Wistar rats (n = 6, 281 ± 13 g body weight) were killed by CO2 asphyxiation and cervical dislocation and the cremaster muscles were surgically removed to ice cold Kreb’s solution. The primary arteriole was micro dissected, cannulated with glass pipettes, placed on the stage of an inverted microscope, pressurised at 70 mmHg (lumen pressure) and superfused with Krebs/HEPES buffer at 34°C. Arterioles were equilibrated for 60 min, until pressure-induced constriction (myogenic tone) developed, usually about 50% of the maximum diameter measured in calcium free Krebs (2 mM, EGTA) and the diameter (μm) was measured by video microscopy using either an automated computer program (DiamTrak) or video callipers as previously described [33]. Changes in diameter were measured in response to increasing concentrations of phenylephrine or acetylcholine (10 nM to 10 μM Sigma, Australia) in the presence and absence of the glucokinase activator RO28-1675 (10 μM). In addition changes in arteriole diameter were recorded in response to increasing concentrations of RO28-1675 or DMSO (vehicle).

Data were collected using MacLab Chart (ADInstruments, v 4.2) and analysed using GraphPad Prism. Results are expressed as the mean ± SEM, n represents the number of arterioles and animals and ANOVA was used to determine the effect of RO28-1675 treatment relative to vehicle (DMSO). Arteriole diameter in the figures is expressed as percentage of the maximum diameter, measured in 0 mM calcium, 2 mM EGTA containing Kreb’s solution.

### Statistical analysis

Data was normalised and shown as the mean ± standard error of the mean (SEM) of three independent experiments performed in triplicate, unless stated otherwise. Western blotting and proteoglycan experiments were analysed by a 1-way ANOVA and glucose utilisation experiments were analysed by 2-way ANOVA. Results were considered significant when the probability was less than 0.05 (*P ≤ 0.05), 0.01 (**P ≤ 0.01) and 0.001(***P ≤ 0.001).

## Results

### Glucokinase activators increase glucose metabolism in MIN6 pancreatic beta cells

**Figure 1:**
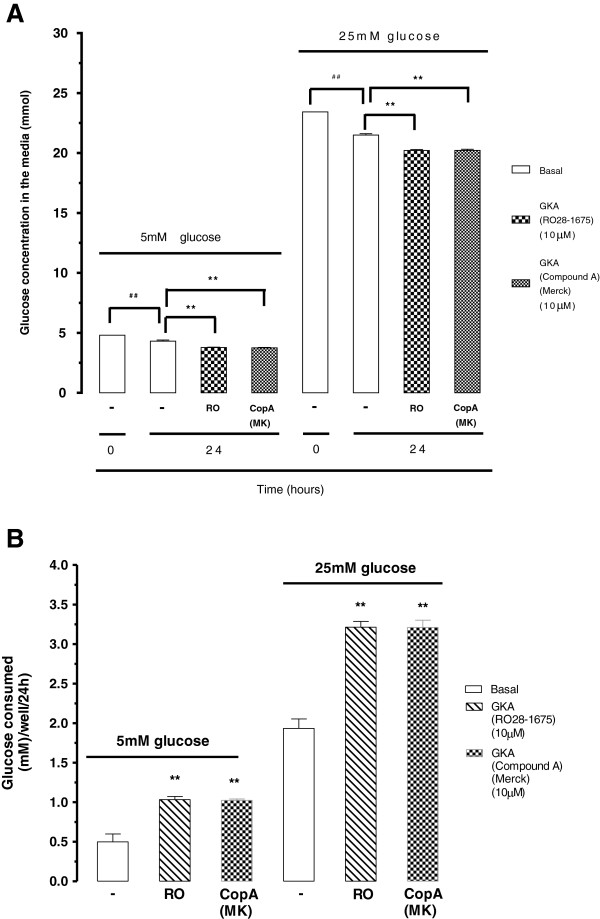
**Effect of GKA on glucose concentration (A) and consumption (B) on rat MIN6 pancreatic beta cells. A**. MIN6 cells treated with either 5 mM or 25 mM glucose medium conditions in the presence and absence of two glucokinase activators (RO28-1675 and Compound A) at 10 μM for 0–24 h. Direct D-glucose levels were measured in the medium for each treatment. Data presented from an experiment utilizing 3 wells/treatment. ##p < 0.01 = basal (24 h) versus basal (0 h) and ##p < 0.01 = treatment versus basal (24 h) determined using one-way-ANOVA on a per well basis. **B**. RO28-1675 and Compound A increased (p < 0.01) the rate of consumption of glucose by MIN6 cells treated with 5 mM and 25 mM glucose compared to basal levels of glucose consumption after 24 h. Results are **p < 0.01treatment versus basal, determined using one-way-ANOVA.

Two GKA compounds, (RO28-1675 and Compound A) were used to test their known action on rat MIN6 pancreatic beta cells expressing GK enzyme. Glucose levels in the medium were measured in the presence and absence of (RO28-1675 and Compound A). The direct measure of glucose concentration in the medium was used to determine glucose consumption. Glucose consumption was calculated by the difference of the basal, RO28-1675 and Compound A after 24 hour incubation under both normal (5 mM) and high (25 mM) glucose conditions. The data for the measurement of the glucose concentration in the media after 24 h inclusion is shown in Figure 1A upper panel. From this data we calculated the actual rate of glucose consumption (Figure 1B). RO28-1675 caused a 106% increase in the rate of glucose consumption/well/24 hour and Compound A caused 104% increase in rate of glucose consumption/well/24 hour in the 5 mM glucose treatment condition (Figure 1B). MIN6 beta cells exposed to 25 mM glucose showed a 286% increase in the rate of glucose consumption compared to 5 mM glucose treatment. Under the 25 mM glucose treatment conditions, both RO28-1675 and Compound A at 10 μM caused 66.3% increase in the rate of glucose consumption/well/24 hour compared to basal (Figure 1B). The data showed that GKAs enhanced the metabolism of glucose in MIN6 pancreatic beta cells and validated the assay for demonstrating the effects of GKAs in cultured cells.

**Figure 2:**
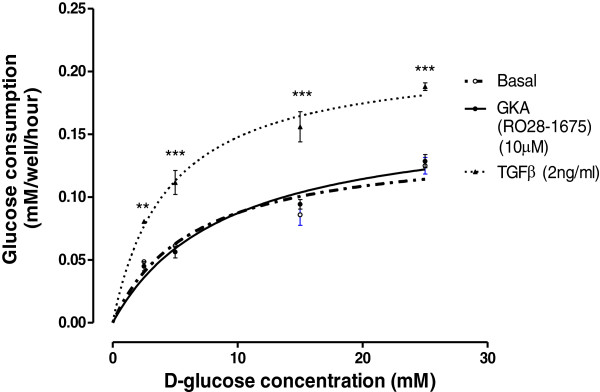
**The relationship between the rate of glucose consumption and D-glucose concentration.** Endothelial cells treated with 2.5, 5, 15, 25 mM D-glucose −/+GKA and TFG-β. Basal represent normal levels of the rate of glucose consumption mM/well/hour in endothelial cells. In the presence of GKA (10 μM) there was no effect on the rate of glucose consumption. Results are the mean ± SEM from 3 experiments in triplicate **p < 0.01 versus basal, ***p < 0.001 versus basal determined using two-way ANOVA.

We then utilised the same assay to assess the effect of GKAs on glucose utilisation by BAEC. BAEC were exposed to different glucose medium concentrations (2.5, 5, 15, 25 mM) +/− GKA (RO28-167 10 μM). Incubation proceeded for different time points (0, 4, 8, 24 h) and glucose levels were measured for each treatment. TGF-β (2 ng/ml), which activates cellular metabolism and increases glucose consumption was used as a positive control for the physiological sensitivity of the assay [34-36]. In the BAEC, the rate of glucose consumption increased with the media glucose concentration reaching a maximum rate of consumption at approximately 30 mM. The rate of glucose consumption was increased in cells treated with TGF-β but was completely unaffected in cells treated with RO28-1675 at the same concentration (10 μM) which stimulated glucose consumption in the MIN6 pancreatic beta cells (compare Figure 1B and Figure 2). The kinetic parameters for the curves are shown in Table 1. The maximum calculated rates of glucose metabolism were 0.14 ± 0.02 mM/well/hour and 0.17 ± 0.02 mM/well/hour (n.s.) in the absence and presence of RO28-1675, respectively and this was increased to 0.21 ± 0.01 mM/well/hour in the presence of TGF-β (Table 1). Half maximally effective concentrations of glucose were not appreciably different amongst the three treatment groups (Table 1). Thus, the GKA RO28-167 did not increase the consumption of glucose by BAECs implying that there is either no GK target or it is expressed at such a low level that its activation by GKAs does not alter glucose utilisation in BAEC.

**Table 1:** **Mean V**_**max**_,**K**_**m **_**and R**^**2 **^**for the rate of glucose consumption in bovine aortic endothelial cells**

	**Basal**	**GKA (RO28-1675) (10 μM)**	**TGF-β (2 ng/ml)**
V_max_	0.14 ± 0.02	0.17 ± 0.02	0.21 ± 0.01
K_m_	6.42 ± 1.97	9.08 ± 2.12	4.47 ± 0.83
R^2^	0.83	0.92	0.91

Results are mean ± SEM of three separate experiments.

### Glucose induced Smad2 activation in endothelial cells

**Figure 3:**
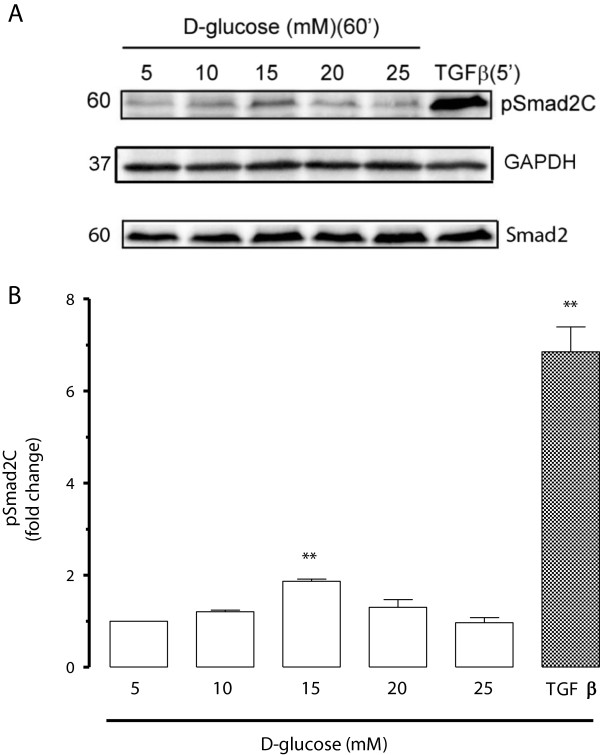
**Effect of D-glucose on the phosphorylation of Smad2C in endothelial cells. A**. Western blot of whole cell lysates from BAECs treated with glucose (5–25 mM) for 60 min. TGF-β (2 ng/ml) for 5 min was used as positive control. Cell lysate were were resolved on SDS-PAGE and transferred onto a PVDF membrane. The membrane was probed with anti-phospho-Smad2(Ser465/467). Also probed with anti-Smad2 and anti-GAPDH. Chemiluminescence was used to detect the proteins of interest. **B**. Histogram is a densitometric quantitation of 3 Western blots. Results **p < 0.01 treatments versus basal (5 mM) n = 3 experiments determined by one-way ANOVA.

**Figure 4:**
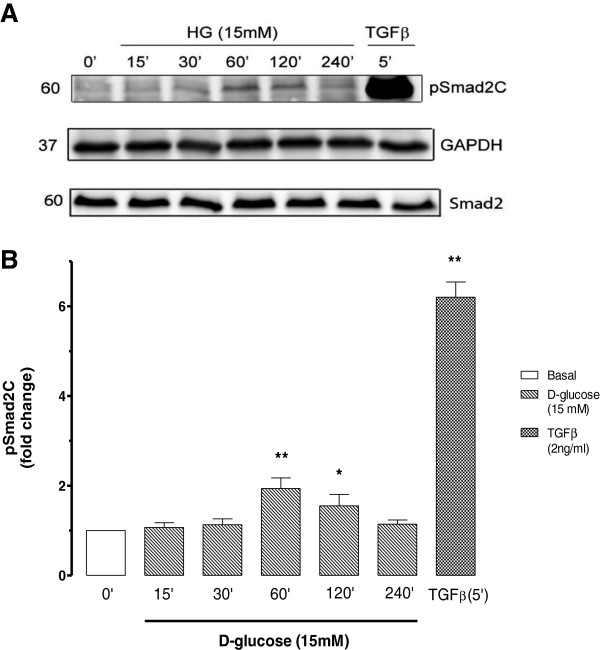
**Effect Smad2C phosphorylation on endothelial cells treated with 15 mM D-glucose for up to 4 h. A**. Western blot of whole cell lysates from BAECs treated with 15 mM D-glucose for up to 240 min. Cells treated with TGF-β (2 ng/ml) (5 min) were used as positive control. Cell lysate were resolved on SDS-PAGE and transferred onto a PVDF membrane. The membrane was probed with anti-phospho-Smad2(Ser465/467) antibody. Also probed with anti-Smad2 and anti-GAPDH. Chemiluminescence was used to detect the proteins of interst. **B**. Histogram is a denstitometric quantitation of. Results determined by one-way ANOVA **p < 0.01 treatments versus basal n = 3 experiments.

High glucose (HG) induces a rapid increase in TGF-β signalling in fibroblasts and epithelial cells [37]. This effect was tested in concentration response studies in vascular endothelial cells. Smad phosphorylation was assessed by Western blotting and the expression of Smad2 and GAPDH were used as controls. BAEC incubated in glucose concentrations (5, 10, 15, 20, 25 mM) for one hour demonstrated that 15 mM induced maximal stimulation of Smad2 transcription factor phosphorylation at the carboxy terminal by 87% increase compared to basal 5 mM (Figure 3A and B). TGF-β (2 ng/ml) used as a positive control caused a 585% increase of Smad2C phosphorylation compared to basal 5 mM (Figure 3). We then evaluated the time dependency of the impact of glucose on phosphoSmad2C levels. BAEC were treated with 15 mM glucose at different time points 0, 15, 30, 60, 120, 240 min (Figure 4A and B). We observed a 94% increase of Smad2C phosphorylation at a peak of 60 min compared to basal levels of Smad phosphorylation at 0 min (Figure 4). TGF-β (2 ng/ml) used as positive control, increased Smad2C phosphorylation by 520% compared to basal levels of Smad phosphorylation. We observed that glucose had a parabolic response in inducing Smad2 phosphorylation in BAEC which was both time and dose-dependent. The peak responses were observed at 15 mM glucose and 60 min stimulation so these conditions were used to study the effects of GKAs on Smad-dependent signalling in BAEC.

### GKAs effects on glucose dependent signalling in endothelial cells

**Figure 5:**
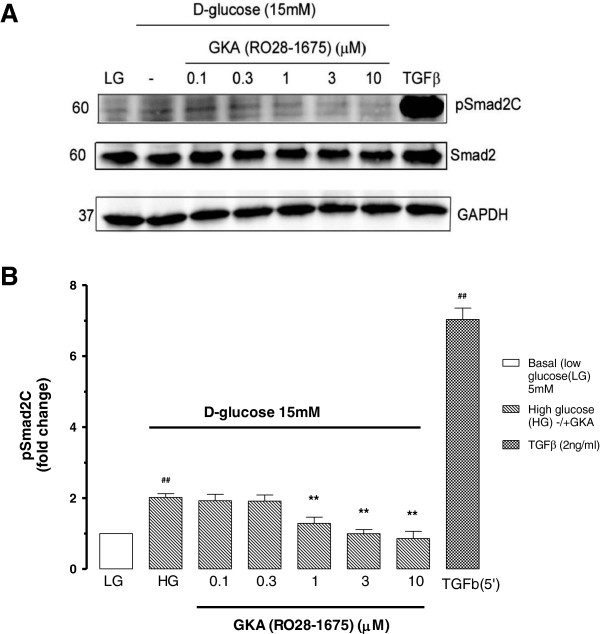
**Effect of GKA on D-glucose mediated Smad2C phosphorylation in endothelial cells. A**. Western blot of whole cell lysates from BAEC treated with D-glucose 15 mM in the presence and absence of GKA (RO28-1675) (0.1-10 μM). TGF-β was used as positive control. Cell lysates were resolved on SDS-PAGE and transferred onto a PVDF membrane. This membrane was probed with anti-phospho-Smad2(Ser465/467). Also probed for anti-Smad2 and anti-GAPDH. Chemiluminescence was used to detect proteins of interest. **B**. Histogram is a densitometric quantitation. Results ##p < 0.01 = compared to untreated basal level (LG) **p < 0.01 compared to agonist treated (HG), n = 4 experiments statistical analysis determined by one-way ANOVA.

We had reasoned that if GKAs increased the effect of glucose on cardiovascular cells, such as endothelial cells, then glucose dependent responses might be intensified. We demonstrated that glucose induces Smad2 carboxy terminal phosphorylation in BAEC indicating activation of the canonical TGF-β signalling pathway in these cells. Accordingly, we tested the effect of a GKA (RO28-1675) on glucose induced Smad2C phosphorylation in BAEC. Cells were treated with glucose (15 mM) +/− GKA (RO28-1675) at concentrations 0.1-10 μM for 60 min and we assessed Smad2C phosphorylation by Western blotting (Figure 5A and B). Glucose (15 mM) increased basal levels of phosphoSmad2C by 101% compared to low glucose (LG) 5 mM (Figure 5). GKA (RO28-1675) caused a concentration-dependent inhibition of 1-10 μM in glucose mediated Smad2C phosphorylation (Figure 5). At the highest concentrations tested (3 μM and 10 μM) GKA restricted the response of HG (15 mM) to basal levels. The half maximally inhibitory concentration of RO28-1675 was approximately 1 μM (Figure 5B). TGF-β (2 ng/ml) used as positive control caused 500% increase of Smad2C phosphorylation levels compared to basal (LG) (Figure 5). This data shows that GKA RO28-1675 caused an inhibition of glucose-dependent phosphoSmad2C signalling in BAECs.

### Effects of GKAs on transforming growth factor beta (TGF-β) stimulated proteoglycan synthesis and specifically glycosaminoglycan elongation

**Figure 6:**
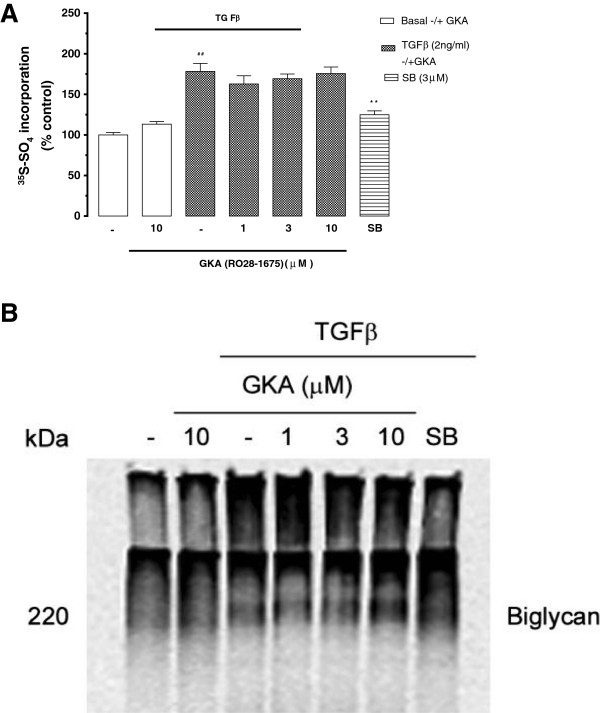
**Effect of GKA on TGF-β mediated **^**35**^**S-SO**_**4 **_**incorporation into proteoglycans by endothelial cells.** Cells treated with TGF-β (2ng/ml) +/-GKA (RO28-1675) 1-10μM for 24 h and SB431542 (3μM) was used as TβR1 antagonist. Media containing secreted proteoglycans was spotted on chromatography paper and CPC precipitated to assess radiolabel incorporation into proteoglycans. Panel **A** shows the quantitation by the CPC precipitation method and Panel **B** shows the resulting SDS-PAGE analysis. Results are mean ±SEM of data normalised to control from three experiments in triplicate, ##p<0.01 versus control and **p<0.01 versus TGF-β using 1-way ANOVA.

The synthesis of proteoglycans (biglycan) in association with increased GAG size is stimulated by the canonical TGF-β Smadphosphorylation pathway in vascular smooth muscle cells and BAEC [38-40]. BAECs were treated with TGF-β (2 ng/ml) to determine the effect on proteoglycan synthesis in the presence and absence of GKA (RO28-1675, 1-10 μM). TGF-β (2 ng/ml) increased ^35^S-SO_4_ incorporation into proteoglycans by 78.2% compared to basal (Figure 6). In the presence of GKA there was no effect on TGF-β mediated ^35^S-SO_4_ incorporation in BAEC. SB431542 (3 μM) is an inhibitor of the serine/threonine kinase activity of TGF-β receptor type 1 (ALK V) [41]; as expected SB431542 (3 μM) almost completely blocked the stimulation by TGF-β (Figure 6). The assessment of proteoglycan size by SDS-PAGE indicated TGF-β (2 ng/ml) increased proteoglycan size and the GKA had no effect on TGF-β stimulated increase in the size of biglycan a response which occurs due to hyperelongation of the lipid binding glycosaminoglycan (GAG) chains (Figure 6) [42-44]. The band with size of 220 kDa corresponds to biglycan [45].

### Effect of GKA on TGF-β mediated Smad2C phosphorylation in BAEC

**Figure 7:**
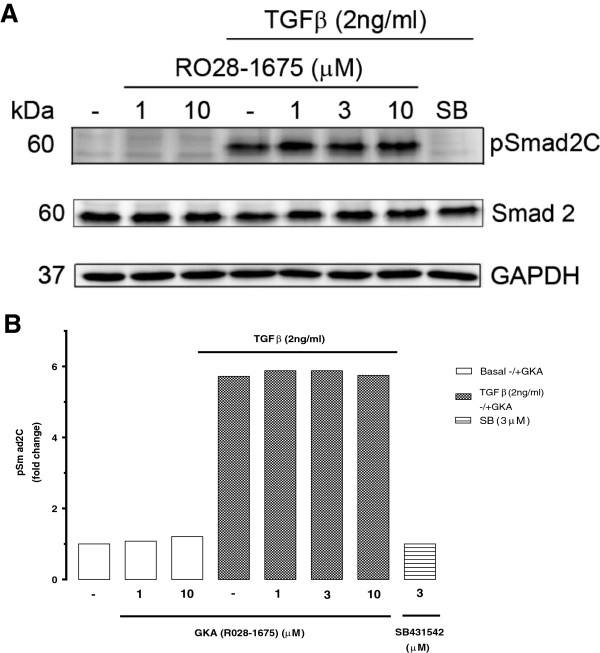
**Effects of GKA (RO28-1675) on TGF-β mediated Smad2C phosphorylation in endothelial cells. A**. Western blot of whole cell lysates from BAECS treated with TGF-β (2 ng/ml) for 5 min in the presence and absence of GKA (RO28-1675) (1-10 μM). SB431542 (3 μM) was used as a positive control. Cell lysates were resolved on SDS-PAGE and transferred onto a PVDF membrane. This membrane was probed with anti-phosphoSmad2(Ser465/467) Also reprobed for anti-Smad2 and anti-GAPDH. Chemiluminescence was used to detect the proteins of interest. **B**. Histogram is a densitometric quantitation of 3 Western blots.

The canonical TGF-β signalling pathway leads to the rapid generation of phosphoSmad2C. To test if GKA had any effects on upstream TGF-β signalling, BAEC were treated with TGF-β (2 ng/ml) for 5 min in the presence of GKA (RO28-1675) (1-10 μM). We observed no effect of GKA on TGF-β stimulated phosphoSmad2 levels (Figure 7). These data indicate that RO28-1657, which blocks glucose dependent Smad signalling does not directly block TGF-β activated Smad signalling in BAECs.

### Direct actions of GKAs on vascular reactivity

**Figure 8:**
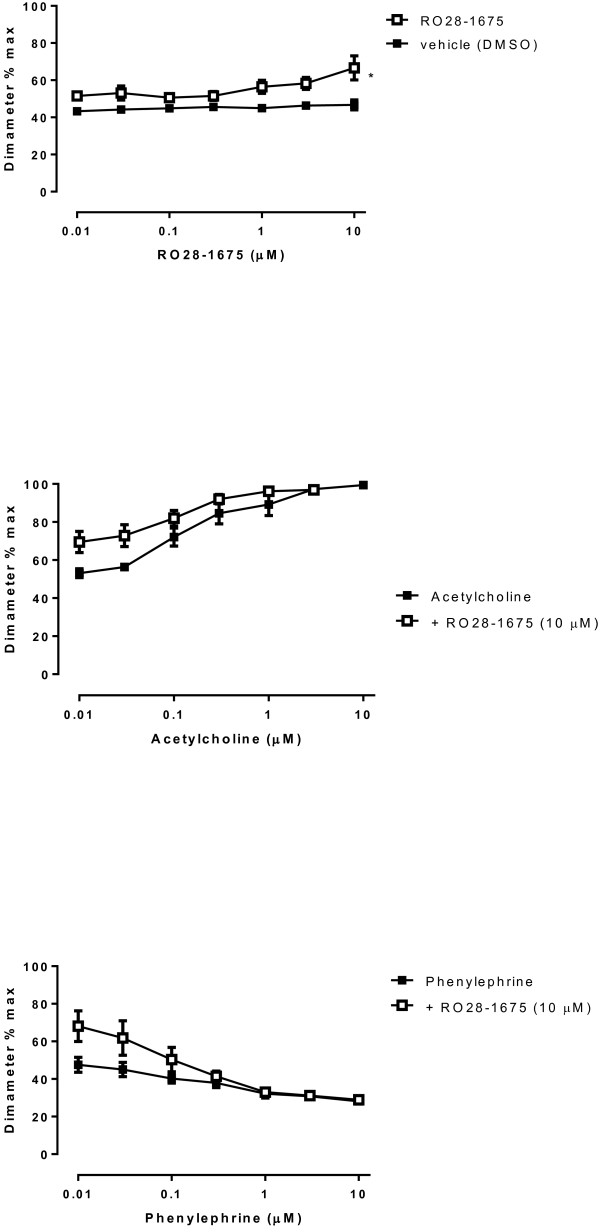
**Changes in pressurised arteriole diameter as responses to increasing concentrations of GKA (RO28-1675).** This figure shows the changes in pressurised arteriole diameter (myogenic tone), expressed as a percentage of the maximum diameter (166 ± 6 μm) measured in 0 mM Ca^2+^/2 mM EGTA buffer. Results are the mean ± SEM from 6 experiments. The upper panel shows the pressurised arteriole diameter responses to increasing concentrations of RO28-1675 or vehicle (DMSO). The * indicates a significant difference (ANOVA, p < 0.05, n = 6) between the RO28-1675 and vehicle treated arterioles. Centre panel, represents the dilatation in response to increasing concentrations of acetylcholine, alone and in the presence of 10 μM RO28-1675. Despite the greater baseline diameter in the presence of 10 μM RO28-1675, the response to acetylcholine was not different. Similarly the lower panel shows the constrictor response to the adrenoceptor agonist phenylephrine, was not different in the presence of 10 μM RO28-1675.

To further explore the effects of GKAs on endothelial and vascular smooth muscle cells, we investigated the direct effects of RO28-1675 in an established assay of vascular tone [33]. Arterioles, maximum lumen diameter 166.0 + 5.6 μm, displayed approximately 50% constriction once equilibrated to a lumen pressure of 70 mmHg at 34°C, dilated maximally in response to acetylcholine (99.3 ± 1.7% at 10 μM) and contracted significantly in the presence of phenylephrine (28.1 ± 1.1% 10 μM). In the presence of the glucokinase activator RO28-1657 arterioles dilated to 66.7 ± 6.5%, at a concentration of 10 mM (P = 0.025, ANOVA, df7, 40). There were no changes in diameter of arterioles treated with the DMSO vehicle. The modest dilation associated with acute RO28-1675 treatment did not alter the contractile sensitivity or maximum response to phenylephrine however it may possibly have increased the sensitivity to acetylcholine mediated dilatation (Figure 8).

## Discussion

We have evaluated the direct vascular effects of several GKAs. We established an islet cell assay that showed GKAs stimulated glucose utilisation and in the same assay in vascular endothelial cells, the drugs had no effect on glucose utilisation but TGF-β (which activates cellular metabolism) used as a positive control did stimulate glucose utilisation. We showed that high glucose in the media stimulated carboxy terminal phosphorylation of the pro-fibrotic transcription factor Smad2 leading to the formation of phosphoSmad2C in a parabolic response for time and concentration. This indicates glucose-mediated regulation of the TGF-β signalling pathway including the activation of TGF-β Type I receptor (TβRI/ALK V). Utilising the optimum stimulatory conditions for high glucose and pSmad2C formation, we showed that the GKA, RO-28-1675, rather than activating phosphoSmad2C formation, actually inhibited high glucose stimulated phosphoSmad2C formation in a concentration dependent manner with a half maximally inhibitory concentration of approximately 1 μM. TGF-β stimulation of the synthesis of the lipid-binding proteoglycan, biglycan, and the elongation of the GAG chains on biglycan represents one component of an in vitro model of atherogenesis because increased GAG chains have enhanced signalling for atherogenic lipoproteins; in this model, RO128-1675 had no effects on TGF-β stimulated biglycan synthesis even at the highest concentration tested (10 μM) so we would conclude that GKAs are silent in this assay in contrast for example to glitazones which are anti-atherogenic [46].

RO128-1675 used as a model GKA, did not affect TGF-β stimulated phosphoSmad2C formation indicating no inhibition of TGF-β signalling. We examined the effect of RO128-1675 on tone of an isolated blood vessel. The GKA slightly relaxed the tissue under resting conditions and this response was lost as the vessel was maximally relaxed with acetylcholine or maximally contracted with phenylephrine. These data demonstrate that a model GKA drug does not have deleterious direct vascular actions in a range of cellular and tissue assays and hence that to the extent that these results can be extrapolated, if reflected *in vivo*, drugs activating GK will not have direct vascular actions which compromise any favourable actions resulting from the glucose lowering action of these drugs. This study clearly indicates that GKAs have no effect on glucose metabolism and atherogenic properties of vascular endothelial cells.

### Role of GKs in glucose homeostasis

Glycaemia is mainly controlled by the glucose mediated release of insulin from the beta cells in the pancreas. The process of entry of glucose across the cell membrane is controlled by hexokinases (HKs) and glucose transporters [19,20]. HK catalyses the initial step of glucose metabolism which is responsible for the conversion of glucose to glucose-6-phosphate (G-6-P) creating a large inward gradient for the entry of glucose to cells via glucose transporters [15]. HK IV, also referred to as glucokinase (GK) is a 50 kDa protein with low affinity and high capacity for glucose and at a physiological concentrations it is not inhibited by its product (G-6-P) [15,16]. GK has so far been found in the liver, pancreatic beta cells, brain (hypothalamus) and gut and it acts as a “glucose sensor” [15,16]. GK plays an important role in pancreatic beta cell insulin secretion [47]. Studies on the importance of GK in insulin secretion, including gain-of-function and loss-of-function studies have shown that other HKI-III isoforms have the ability to compensate for the loss of GK; in pancreatic GK knockout mice insulin release was still occurring in the absence of the GK enzyme [16,48]. Also, liver HK knockout mice showed that glucose homeostasis was still maintained [16]. In the absence of GK enzyme in the pancreas and the liver, other HKs can take over the action of GK [16,48].

### Hyperglycaemia and the complications of diabetes

The hyperglycaemia of diabetes has deleterious effects on the microvasculature and on balance negative effects on the macrovasculature. Therapeutically lowering blood glucose levels can have beneficial effects on microvasculature but the results are less clear for the macrovasculature. GKAs are a class of drugs which stimulate the uptake of glucose into cells. In the setting of diabetes this is most relevant in islet and liver cells. In islet cells, the stimulation of glucose uptake leads to enhanced insulin secretion and an overall hypoglycaemic effect being the mode of action of these drugs in Type 2 diabetes. However, if glucose causes damage to vascular cells then the stimulation of glucose uptake by GKAs might be expected to increase the glucose toxicity and oppose any action resulting from a reduction in glycaemia.

Thus, GKAs might have deleterious actions due directly to their target being present in vascular endothelial cells and their mode of action increasing the effect of glucose with resulting deleterious effects. Additionally, many drugs have pleiotropic actions, often toxic actions unrelated to the prime mechanism of action of the index drug so we conducted experiments which evaluated the vascular actions of GKAs based on both of these scenarios. We established an assay that could demonstrate the action of a GKA to stimulate glucose uptake and utilisation in an islet cell line. Using the same experimental model we found that a GKA had no effect on glucose uptake and utilisation by vascular endothelial cells. These experiments exclude the possibility that GKAs might cause vascular toxicity by a mechanism which is inherent in their anti-hyperglycaemic action in Type 2 diabetes. We then investigated the direct actions of GKAs on vascular cells and on intact blood vessels.

### Hypoglycaemic actions of GKAs

GKA (RO28-1675) and Compound A are two members of anti-hyperglycemic GKA family of pre-clinical therapeutic agents. They act by increasing the activity of glucose-phosphorylating enzyme GK in pancreatic beta cells leading to increased glucose uptake and insulin production and secretion [10]. We have confirmed the known mechanism of action of GKAs on rat MIN6 pancreatic beta cells expressing GK enzyme and found that GKAs (RO28-1675) and Compound A both appreciably increased the cellular utilization of glucose by MIN6 cells. Thus, GKAs stimulated GK enzyme in the MIN6 cells and caused an increase in the rate of glucose consumption. The presence and expression of glucokinase isoforms in vascular cells and tissues has not been extensively studied. Our assay of increased glucose utilisation assessed by depletion of glucose from the culture media over several hours is essentially a functional assay for the presence of a target in vascular cells which is stimulated by GKA drugs. That we found no effect of the GKA on glucose uptake in vascular endothelial cells indicates that a GKA sensitive isoform is not present in these cells or at least is not present at a level that could increase the exposure of cells to glucose. It is not unusual for therapeutic targets to be present in non-target tissues however it has not always been investigated. In the case of thiazolidinediones there was conflicting evidence as to the level and importance of the expression of PPAR-α [49] and PPAR-γ [50] in vascular smooth muscle cells and hence the contribution that such targets may make to the vascular actions of these drugs [51]. Our data allows for a favourable understanding that GKAs do not intensify the deleterious actions of glucose on vascular endothelial cells.

### Fibrotic actions of glucose via Smad transcription factors – inhibition by GKAs

Recent published data shows that exposure of fibroblasts and epithelial cells to elevated levels of glucose enhances the activation of transforming growth factor TGF-β Type I receptor (TβRI)/Smad3 signalling and mediates the development of cellular hypertrophy [37]. In concurrence with these finding, our data clearly shows that HG can induce Smad signalling in BAEC. Western blotting analysis for carboxy terminal phosphorylated Smad2, revealed that glucose induced Smad2C phosphorylation with parabolic responses for both time and concentration. The HG induced Smad activation via the TGF-β signalling pathway is also supported by other studies [37]. Smad3 activation was observed in these cells in presence of HG demonstrating that Smad phosphorylation results from activation of autocrine TGF-β signalling [52-54]. It is noteworthy that the phosphoSmad2C response in endothelial cells peaked in the pathophysiological range for glucose in people with Type 2 diabetes. Although the response declined at high concentration and longer time points it would be a reasonable assumption that regularly and widely varying glucose levels throughout the day in person with Type 2 diabetes might be associated with fluctuating levels of phosphorylated Smad and Smads are well known to be associated with fibrotic states such as kidney disease. The significance of this data is that the outcome of TGF-β receptor signalling in vascular cells is associated with *de novo* synthesis of collagen and plasminogen activator inhibitor 1 (PAI-1) which are well known fibrotic and pro-coagulant characteristics of the diabetic milieu [55-58]. This data provided the basis for our experiments to test if GKA could intensify a glucose dependent response such as Smad2C phosphorylation in endothelial cells.

We showed that GKA (RO28-1675) caused concentration dependent inhibition of glucose stimulated Smad2C phosphorylation levels in endothelial cells. This effect is independent of any action of HK because GKA does not affect the HK enzyme in endothelial cells. GKA has caused a pleiotropic action, on glucose dependent signalling unrelated to the known mechanism of action, which can further be investigated. This may be a favourable pleiotropic action because the GKA agent decreased the phosphorylation of Smad2C a response which is clearly pro-fibrotic in many tissues [55,58].

### Direct effects of GKAs on vascular proteoglycan synthesis

We have studied the effect of GKA on proteoglycan, specifically biglycan synthesis, which was used as a model for the initiation of atherosclerosis [42,44,59]. TGF-β is known to increase the length of GAG chains on proteoglycans in endothelial cells [39]. Our aim was to further characterise the TGF-β mediated proteoglycan synthesis specifically glycosaminoglycan elongation in presence of GKA (RO28-1675). Our results showed that TGF-β increased proteoglycan synthesis, specifically GAG elongation but the GKA had no effect on radiolabeled sulfate incorporation into proteoglycans and does not play a role in TGF-β mediated GAG elongation in BAECs. This is in contrast to a range of anti- hyperglycaemic agents which do inhibit growth factor stimulated proteoglycan synthesis but much of this data is in vascular smooth muscle as opposed to the endothelial cells employed in the current studies [60,61].

Since GKA (RO28-1675) blocked glucose dependent Smad2 phosphorylation but not the direct effects of TGF-β on proteoglycan synthesis in BAEC we looked directly at the effects of RO28-1675 on upstream TGF-β signalling in the BAECs. We investigated the effect of GKA (RO28-1675) on TGF-β mediated Smad2C phosphorylation in endothelial cells. RO28-1675 had no effect on TGF-β mediated Smad2C phosphorylation even at the highest concentration tested which had shown effects in other assays. This data has demonstrated that RO28-1675 has no effect on TGF-β signalling in endothelial cells.

## Conclusions

In conclusion, the new and emerging class of anti-hyperglycemic agents, glucokinase activators, have been investigated for their actions on vascular endothelial cells and intact blood vessels. We have demonstrated that there is no effect of GKAs on glucose utilisation by endothelial cells. A GKA had an inhibitory effect on glucose stimulated Smad phosphorylation an effect that must be independent of glucose metabolism because GKAs do not affect glucose metabolism in endothelial cells. We demonstrated that glucose stimulated Smad phosphorylation in a time and concentration dependent manner and this response, which might underpin some of the vascular glucotoxicity, was surprisingly blocked by a GKA.

This unexpected action of GKAs, if long lasting and occurring *in vivo*, a parameter which is unknown at this time, will tend to be beneficial in the context of the processes causing diabetes vascular complications. Overall, we observed no effects of GKAs which would compromise the use of this class of drug treating the hyperglycaemia of Type 2 diabetes and based on this work the ultimate clinical utility will depend upon their long term anti-hyperglycaemic actions and the extent to which this translate into reduced cardiovascular disease.

## Abbreviations

BAECs: Bovine aortic endothelial cells; ECs: Endothelial cells; GKAs: Glucokinase activators; GK: Glucokinase; G-6-P: Glucose-6-phosphate; GAG: Glycosaminoglycan; HK: Hexokinases; HG: High glucose; LG: Low glucose; PAI-1: Plasminogen activator inhibitor 1; SDS-PAGE: Sodium dodecyl sulfate polyacrylamide gel electrophoresis; TGF-β: Transforming growth factor beta; ALK V: TGF-β receptor type 1.

## Competing interests

The authors declare that they have no competing interests.

## Authors’ contributions

PJL conceived the study and with NO developed the experimental program, designed the experiments and interpreted the results and drafted the manuscript. DK assisted with the preparation and interpretation of the experimental results and with the preparation of the manuscript. SA undertook most of the experiments under the laboratory supervision of RG. SJP and RG prepared the figures. WZ provided technical expertise around the biochemical assays and assisted PJL in the development of the research strategy. SJP undertook and interpreted the vessel experiments. NC and DG provided the clinical pharmacy and medical input to set the context for the interpretation of the experiments and the relationship of the project to the therapeutics of the cardiovascular complications of diabetes. All authors read and approved the final manuscript.
